# Sharp increase of imported *Plasmodium vivax* malaria seen in migrants from Eritrea in Hamburg, Germany

**DOI:** 10.1186/s12936-016-1366-7

**Published:** 2016-06-17

**Authors:** Louise Roggelin, Dennis Tappe, Bernd Noack, Marylyn M. Addo, Egbert Tannich, Camilla Rothe

**Affiliations:** Section of Tropical Medicine and Infectious Diseases, Department of Medicine, University Medical Centre Hamburg-Eppendorf, Hamburg, Germany; Bernhard Nocht Institute for Tropical Medicine, Hamburg, Germany; German Centre for Infection Research, partner site Standort Hamburg-Lübeck-Borstel, Hamburg, Germany

**Keywords:** Malaria, *Plasmodium vivax*, Refugees, Eritrea, Horn of Africa

## Abstract

**Background:**

Since 2014, a considerable increase in *Plasmodium vivax* malaria has been observed in Germany. The majority of cases was seen in Eritrean refugees.

**Methods:**

All patients with *P. vivax* malaria admitted to the University Medical Centre Hamburg-Eppendorf Germany from 2011 until August 2015 were retrospectively identified by the hospital coding system and data was matched with records from the laboratory diagnostics unit of the Bernhard Nocht Institute for Tropical Medicine, Hamburg, Germany.

**Results:**

Between May 2014 and August 2015, 37 cases were reported in newly-arrived Eritrean refugees at the University Medical Centre Hamburg-Eppendorf, Germany. Relapses occurred due to difficulties in procurement of primaquine.

**Conclusion:**

Countries hosting Eritrean refugees need to be aware of vivax malaria occurring in this group and the risk of autochthonous cases due to local transmission by indigenous, vector competent Anopheles species.

## Background

Around 500–600 annual cases of malaria were reported in Germany in the years before 2013. 80 % were *Plasmodium falciparum* malaria, almost exclusively imported from West Africa [1]. Other forms of malaria were of minor importance (7 % vivax malaria in 2013). Since 2014, this has changed dramatically: the number of reported malaria cases has almost doubled compared to the previous years [1] and clinicians are observing growing numbers of *Plasmodium vivax* infections, predominantly in refugees from Eritrea. *Plasmodium vivax* infections are characterized by relapses of malaria due to persistent liver stages of the parasite (hypnozoites). Those relapses can be prevented currently only by 8-aminoquinoline anti-malarials, such as primaquine [2].

In Europe, the number of asylum seekers status is continuously climbing with more than 200,000 applications in the European Union in 2014, most of them in Germany [3, 4]. In the previous 2 years, there was a sharp increase in the number of refugees and migrants crossing the Mediterranean Sea, with the largest group coming from Eritrea and Syria [3, 5].

In this report, the impact of these recent migration trends on the epidemiology of malaria cases seen in a large referral and teaching hospital in Germany is described.

## Methods

The study was performed at the University Medical Centre Hamburg-Eppendorf (UKE), a 1600-bed tertiary-care referral and teaching hospital with a specialist tropical medicine unit, in Hamburg, northern Germany. For tropical diseases the catchment area of this hospital is northern Germany. All patients admitted to the hospital with *P. vivax* malaria from 2011 until August 2015 were retrospectively identified by the “International Statistical Classification of Diseases and Related Health Problems” (ICD) and matched the data with records from the laboratory diagnostics unit of the Bernhard Nocht Institute for Tropical Medicine, Hamburg, which is the national reference laboratory for tropical pathogens in Germany. Diagnosis of malaria was performed by reading thin and thick blood films. In cases of low parasitaemia, blood was subjected to species-specific polymerase chain reaction (PCR) for species differentiation. The success of subsequent primaquine therapy after initial anti-malarial chemotherapy was evaluated by telephone interviews of patients or their legal guardians and by reviewing hospital documents.

Statistical analysis was performed using SPSS software (SPSS inc., version 20).

This study has been granted an exemption from requiring ethics approval as it is an retrospective study and all patient information have been directly anonymised. The exemption was granted by be ethics committee of the physician Chamber Hamburg, Germany.

## Results

**Fig. 1:**
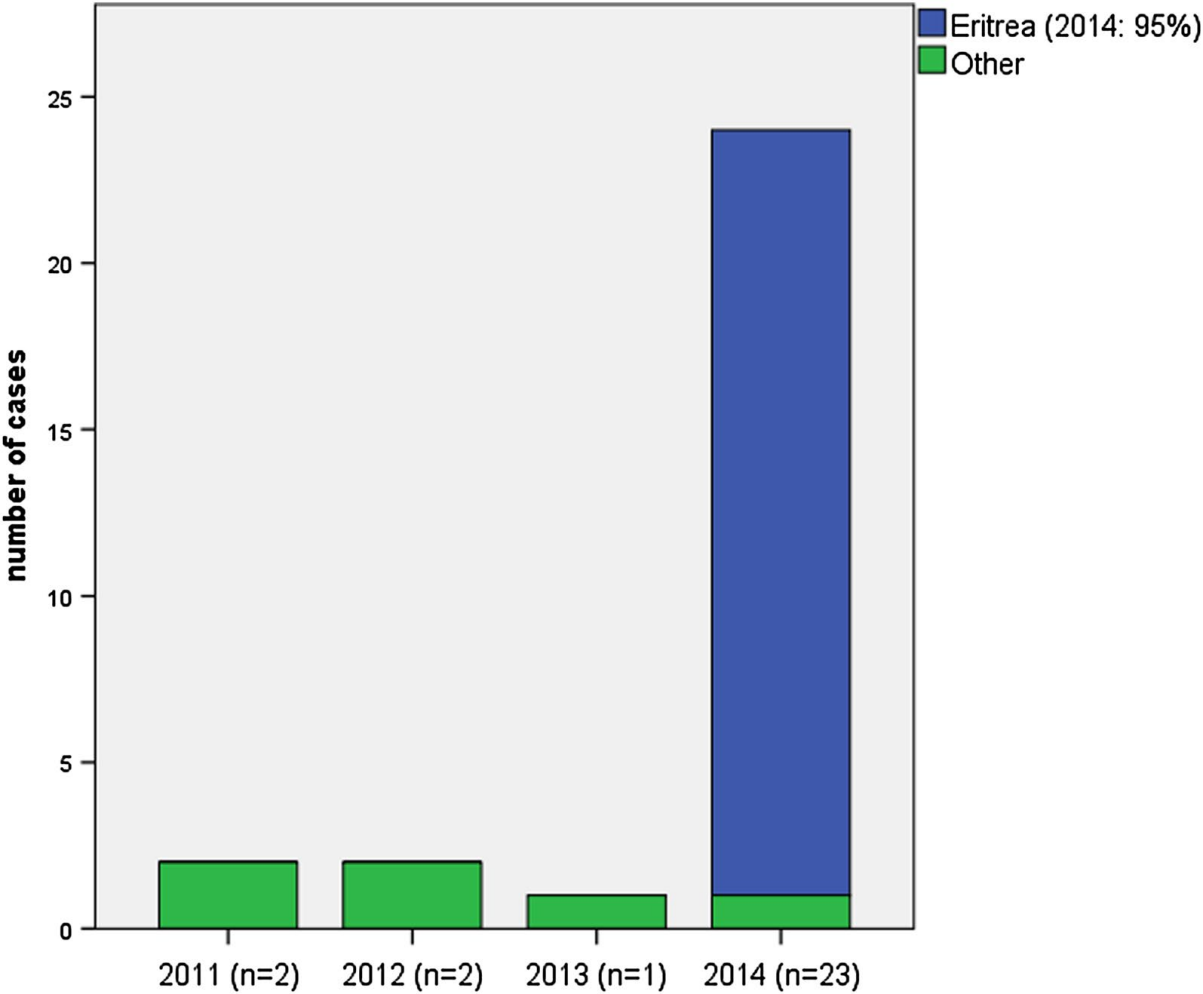
Number of cases *of P. vivax* malaria reported at the UKE Hamburg, Germany by country of origin from 2011 to 2014

From 2011 to 2013, 200 cases of malaria were treated at the UKE, 90 % of them were due to infection with *P. falciparum*. *Plasmodium vivax* accounted for only one or two cases per year, imported from Afghanistan and Benin (2011), Pakistan and Brazil (2012) or Vanuatu (2013). In 2014, a sharp increase was recognized and 23 cases of vivax malaria were diagnosed, of which 22 occurred in Eritrean refugees and one case in a refugee from Syria. In 2015, until August, there were 16 cases of vivax malaria, 15 cases in Eritrean refugees and one case in a returning traveller from Indonesia. One additional case was an unspecified tertian malaria from Uganda (Fig. 1).

**Fig. 2:**
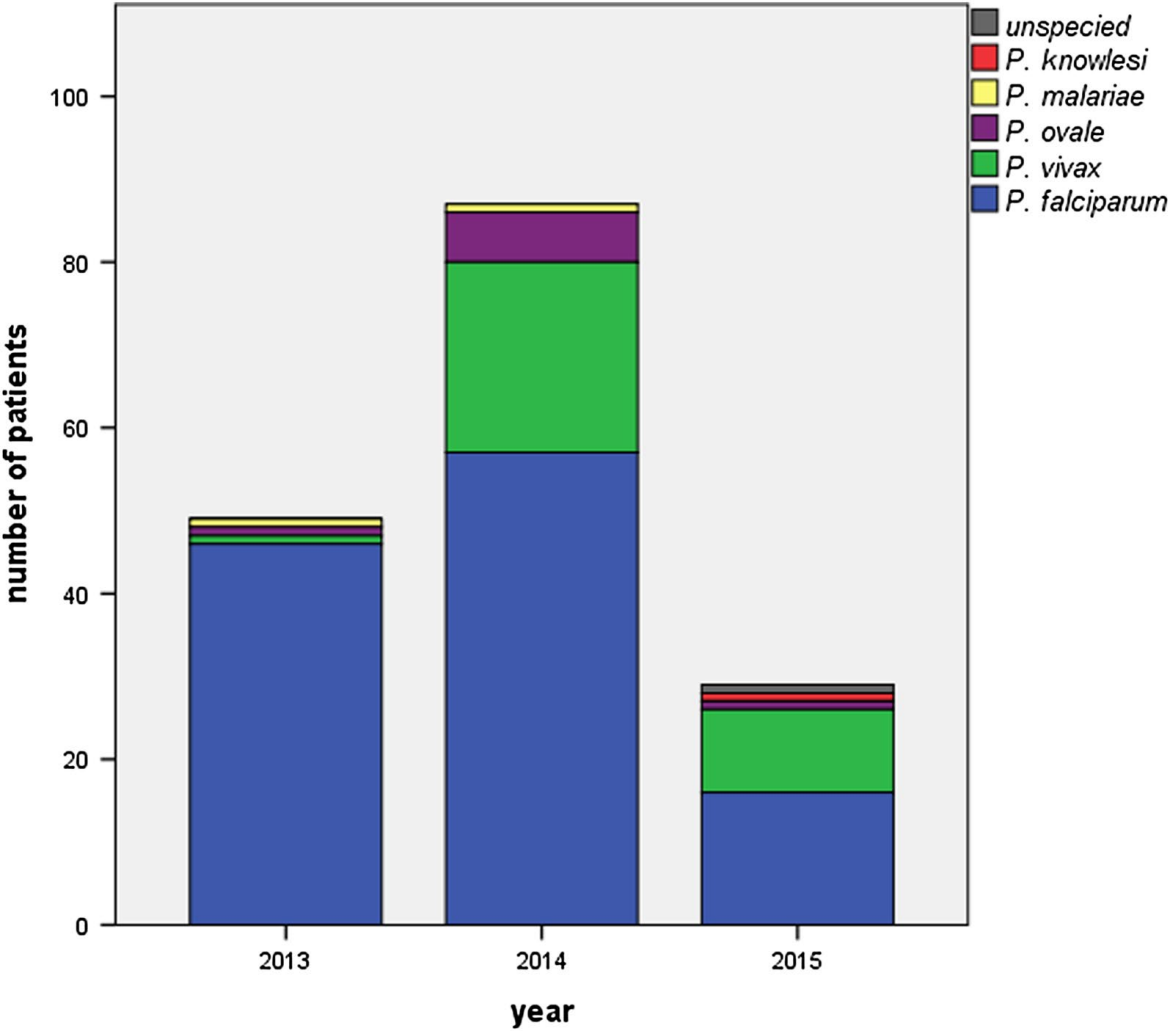
Malaria cases stratified by *Plasmodium* species treated at the UKE: n = 49, 2014: n = 87, 2015: n = 29 (until June 2015 only)

There was no concomitant increase in *P. falciparum* cases (2011: 59, 2012: 55, 2013: 46, 2014: 57, 2015: 55). Therefore, a relative increase of vivax malaria from only 2 % of all cases in 2013 to 26 % in 2014 and 34 % in 2015 was observed (Fig. 2).

**Fig. 3:**
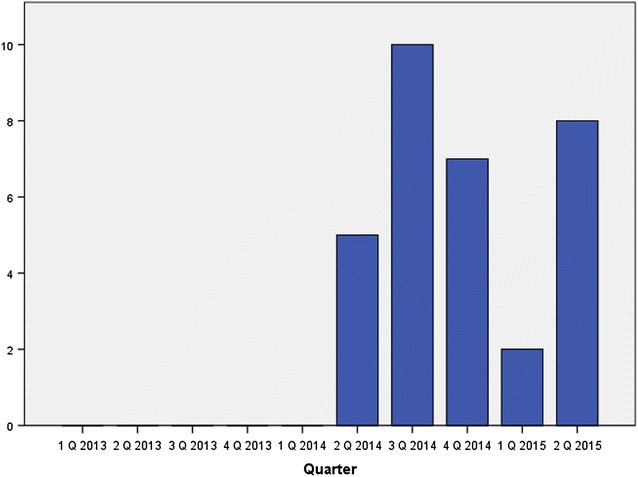
Number of *P. vivax* cases in newly arrived Eritrean refugees treated at the UKE from January 2013 until June 2014 (n = 32)

Of the 37 Eritrean patients seen in the institution since 2014 (Fig. 3), 34 were male (92 %), median age was 19 years (range 12–37 years), and 35 % (13 patients) were of minor age (<18). One patient had a mixed infection with *P. falciparum*. One additional case, not included in the following statistics, had an infection with *Plasmodium ovale*. For 35 out of the 37 *P. vivax* patients, the date of arrival to Germany was known. Of those, all had arrived to Germany during the previous 6 months (median 2 weeks, range 1–180 days). All reported migration through Ethiopia and/or Sudan before embarking from Libya to Europe on the “Central Mediterranean Route” [5].

For 25 patients information about previous presumptive malaria attacks was available, 22 of these reported having had at least one episode of fever during their journey from Eritrea, 16 received some anti-malarial treatment during their journey. In six cases, a relapse was noticed after recent hospitalization and anti-malarial therapy in Germany, even though in all of these cases subsequent primaquine therapy had been recommended upon discharge to prevent further relapses.

Patients were treated with atovaquone/proguanil (30 cases), artemether-lumefantrine (4 cases) or chloroquine (3 cases). For all patients subsequent primaquine therapy was recommended. One patient had glucose-6-phosphate dehydrogenase (G6PD) deficiency. In 18 cases it was possible track down if the therapy with primaquine was taken: In 14 of these (78 %) primaquine was administered as recommended. The remaining patients were lost to follow up.

## Discussion

Until 2013, only very few malaria cases seen at our University Medical Centre were due to infection with *P. vivax*. This changed considerably in 2014, when more than a quarter of cases were diagnosed as vivax malaria; the trend currently continues in 2015 and was also observed on a national level [1]. The increasing number of malaria cases coincides with increasing numbers of asylum seekers from Eritrea [4] and most vivax cases occurred in this group. Thus, Eritrea has become the most common country of imported malaria cases seen at the UKE in Hamburg, in 2014 and 2015.

Data from Sweden, where Eritrean refugees were the third largest group of refugees in 2014 also, indicate a similar development [6]. Interestingly, data from Tel Aviv suggest that a similar phenomenon already took place in Israel in 2010 [7], when there were still only small numbers of refugees from Eritrea in Germany that did not seem to impact malaria epidemiology in the country.

It remains unclear if the infection was acquired at home in Eritrea or during the journey. Interestingly, one case of tertian malaria was observed, which was microscopically most likely an infection with *P. ovale*. Unfortunately, a specimen diagnosis by PCR was not possible, since no blood sample was left in this case. Another *P. ovale* infection imported from East Africa seen at another hospital was, however, confirmed by PCR.

*Plasmodium falciparum* and *P. vivax* predominate in Eritrea (60 and 39 %), Ethiopia (64 and 36 %) and Sudan (95 and 5 %, respectively) [8], whereas *P. ovale* is most often seen in travellers and expatriates returning from West Africa. Nevertheless, data published in 2013 reported *P. ovale* infections in neighbouring north-west Ethiopia [9], the parasite therefore seems to be endemic in the region. West African migrants from a region where *P. ovale* is fairly common and East African migrants both use the route via Libya to come to Europe [5]. It is, therefore, also possible that a transmission between migrants may occur in countries like Libya where waves of migrating refugees converge and competent vectors are present.

Furthermore, it is important to note that *Anopheles* species competent for *P. vivax* transmission are present in most European countries, such as *Anopheles atroparvus* in Germany. Thus, the increased introduction of *P. vivax* into Europe poses the risk for local transmission and the occurrence of autochthonous malaria, as recently reported from Greece [10].

It is of concern that there were a number of relapses of vivax malaria in patients with previous inpatient treatment in Germany, despite the fact that subsequent therapy with primaquine had been recommended. Primaquine is currently not licensed in Germany and has to be ordered from abroad. This makes prompt primaquine treatment challenging, especially for patients currently seeking asylum who may not have a fixed place of residence.

## Conclusion

A steep rise in vivax malaria in was observed at the University Medical Centre Hamburg-Eppendorf Germany since May 2014, almost exclusively seen in Eritrean refugees. Awareness of possible *P. vivax* infection in this group of patients and correct diagnosis is of major importance in order to treat the patients effectively by successive primaquine administration. This is of considerable importance to prevent relapses and to minimize the risk of local transmission.
